# (3*R*,6*R*,12*R*,20*S*,24*R*)-20,24-Ep­oxy­dammarane-3,6,12,25-tetra­ol

**DOI:** 10.1107/S1600536811008609

**Published:** 2011-03-12

**Authors:** Lei Zhang, Huan-Mei Guo, Wen-Juan Li, Yi-Jun Gao, Qing-Guo Meng

**Affiliations:** a School of Pharmacy, Yantai University, Yantai 264005, People’s Republic of China; b Microscale Science Institute, Weifang University, Weifang 261061, People’s Republic of China

## Abstract

In the title compound, C_30_H_52_O_5_, the three six-membered rings are in chair conformations, the five-membered ring is in an envelope form and the tetra­hydro­furan ring has a conformation inter­mediate between half-chair and sofa. Intra­molecular O—H⋯O hydrogen bonds may influence the conformation of the mol­ecule. In the crystal, mol­ecules are linked by inter­molecular O—H⋯O hydrogen bonds, forming a three-dimensional network.

## Related literature

The title compound was prepared from 20(*S*)-protopanaxatriol which was degraded from *Panax quinquefolium* saponin. For background to and the medicinal properties of *Panax ginseng* and *Panax quinquefolium*, see: Shibata *et al.* (1985 ▶); Takano *et al.* (1999 ▶); Yu *et al.* (2007 ▶); Wang *et al.* (2010 ▶). For related structures, see: Shi *et al.* (1992 ▶); Meng *et al.* (2010 ▶).
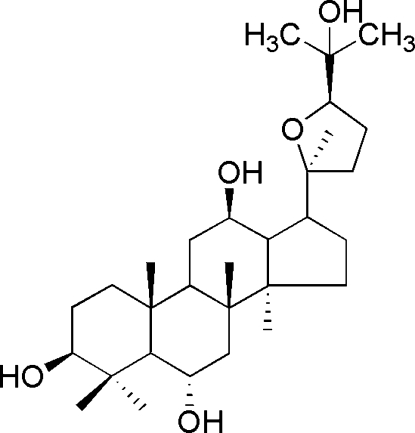


## Experimental

### 

#### Crystal data

C_30_H_52_O_5_
*M* *_r_* = 492.72
Orthorhombic, 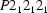

*a* = 12.7918 (6) Å
*b* = 13.7842 (7) Å
*c* = 16.0902 (8) Å
*V* = 2837.1 (2) Å^3^
*Z* = 4
Mo *K*α radiation
μ = 0.08 mm^−1^
*T* = 298 K
0.54 × 0.50 × 0.50 mm

#### Data collection

Bruker SMART CCD diffractometer
16320 measured reflections
3141 independent reflections
2911 reflections with *I* > 2σ(*I*)
*R*_int_ = 0.020

#### Refinement

*R*[*F* ^2^ > 2σ(*F* ^2^)] = 0.042
*wR*(*F* ^2^) = 0.113
*S* = 1.05
3141 reflections
325 parameters
6 restraints
H-atom parameters constrained
Δρ_max_ = 0.26 e Å^−3^
Δρ_min_ = −0.24 e Å^−3^

### 

Data collection: *SMART* (Bruker, 1997 ▶); cell refinement: *SAINT* (Bruker, 1997 ▶); data reduction: *SAINT*; program(s) used to solve structure: *SHELXS97* (Sheldrick, 2008 ▶); program(s) used to refine structure: *SHELXL97* (Sheldrick, 2008 ▶); molecular graphics: *SHELXTL* (Sheldrick, 2008 ▶); software used to prepare material for publication: *SHELXTL*.

## Supplementary Material

Crystal structure: contains datablocks global, I. DOI: 10.1107/S1600536811008609/lh5198sup1.cif

Structure factors: contains datablocks I. DOI: 10.1107/S1600536811008609/lh5198Isup2.hkl

Additional supplementary materials:  crystallographic information; 3D view; checkCIF report

## Figures and Tables

**Table 1:** Hydrogen-bond geometry (Å, °)

*D*—H⋯*A*	*D*—H	H⋯*A*	*D*⋯*A*	*D*—H⋯*A*
O1—H1*A*⋯O2	0.82	2.41	2.882 (3)	117
O2—H2⋯O5	0.82	1.95	2.697 (2)	152
O3—H3⋯O4^i^	0.82	2.12	2.929 (3)	169
O4—H4⋯O2^ii^	0.82	2.13	2.890 (2)	153

Symmetry codes: (i) 
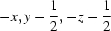
; (ii) 
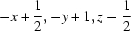
.

**Fig. 1.**
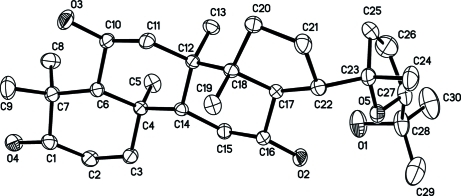
The molecular structure of the title compound showing ellipsoids drawn at the 30% probability level. H atoms are not shown.

## supplementary crystallographic information

### Comment

Both Panax ginseng and Panax quinquefolium, belonging to Araliaceae, are well known traditional medicinal herbs. They are used as tonics and for the treatment for diseases, such as tumor and myocardial ischemia. Panax ginseng contains numbers of saponins,  namely ginsenoside and an oleanolic acid-type saponin in addition to the major protopanaxadiol and protopanaxatriol-type saponins (Shibata *et al.*,1985). Panax quinquefolium contains an ocotillol-type (20*S*, 24*R*-epoxyside) saponin with high anti-tumor activity (Takano *et al.*,1999), as well as oleanolic acid-type, protopanaxadiol and protopanaxatriol-type saponins. (3*R*,6*R*,12*R*,20S,24*S*)-20,24-Epoxy-dammarane-3,6,12,25-tetraol and (3*R*,12*R*,20*S*,24*R*)-20,24-epoxy-dammarane-3,12,25-triol are found to possess cardioprotective effect on myocardial injury induced by isoproterenol in rats (Yu *et al.*,2007; Wang *et al.*, 2010). As part of our ongoing investigation of ocotillol-type compounds and their cardioprotective effect on myocardial injury, we report herein the crystal structure of the title compound, (I).

The molecular structure of (I) is shown in Fig. 1. In the molecule, all bond lengths and angles are within normal ranges (Shi *et al.*,1992; Meng *et al.*, 2010). The six-membered rings A(C11,C13,C16,C17,C18,C19), B(C13,C15,C16,C22,C32,C33), and C(C22,C24,C25,C26,C29,C32) are in chair conformations. Ring D(C8,C9,C10,C11,C19) has an envelope form with atom C11 forming the flap. The tetrahydrofuran ring has a conformation intermediate between half-chair and sofa forms. In the crystal, molecules are linked by O—H···O hydrogen bonds (Table 1) to form a three-dimensional network.

### Experimental

20(S)-protopanaxatriol was degraded from Panax quinquefolium saponin with sodium in glycerine at about 473 - 503K and seperated by silica flash chromatography. (3*R*,6*R*,12*R*,20S,24*R*)-20,24-Epoxy-dammarane-3,6,12,25-tetraol was synthesized from 20(S)-protopanaxatriol in the presence of *N*,*N*-dimethylaminopyridine, pyridine and acetic anhydride. The esters were oxidized by *m*-CPBA and the title compound was obtained by saponification with sodium hydroxide in DMSO and seperated by silica flash chromatography. Finally, the crystals were dried at room temperature the title compound was crystallized from ethyl acetate. Single crystals suitable for X-ray measurements were obtained by recrystallization of an acetone solution of the title compound at room temperature.

### Refinement

In the absence of significant anomalous dispersion effects the Friedel pairs were merged. All H atoms were fixed geometrically and allowed to ride on their attached atoms, with C—H distances in the range 0.93–0.97 Å; O—H = 0.86Å and with *U*_iso_(H) = 1.2*U*_eq_(C) or 1.5*U*_eq_(C_methyl_,O). The absolute configuration is based on unchanging stereochemical centers in the synthesis

### Crystal data

**Table:**

C_30_H_52_O_5_	*F*(000) = 1088
*M**_r_* = 492.72	*D*_x_ = 1.154 Mg m^−^^3^
Orthorhombic, *P*2_1_2_1_2_1_	Mo *K*α radiation, λ = 0.71073 Å
Hall symbol: P 2ac 2ab	Cell parameters from 8789 reflections
*a* = 12.7918 (6) Å	θ = 2.2–28.0°
*b* = 13.7842 (7) Å	µ = 0.08 mm^−^^1^
*c* = 16.0902 (8) Å	*T* = 298 K
*V* = 2837.1 (2) Å^3^	Block, colourless
*Z* = 4	0.54 × 0.50 × 0.50 mm

### Data collection

**Table:**

Bruker SMART CCD diffractometer	2911 reflections with *I* > 2σ(*I*)
Radiation source: fine-focus sealed tube	*R*_int_ = 0.020
graphite	θ_max_ = 26.0°, θ_min_ = 2.0°
φ and ω scans	*h* = −14→15
16320 measured reflections	*k* = −16→17
3141 independent reflections	*l* = −17→19

### Refinement

**Table:**

Refinement on *F*^2^	Primary atom site location: structure-invariant direct methods
Least-squares matrix: full	Secondary atom site location: difference Fourier map
*R*[*F*^2^ > 2σ(*F*^2^)] = 0.042	Hydrogen site location: inferred from neighbouring sites
*wR*(*F*^2^) = 0.113	H-atom parameters constrained
*S* = 1.05	*w* = 1/[σ^2^(*F*_o_^2^) + (0.0809*P*)^2^ + 0.2644*P*] where *P* = (*F*_o_^2^ + 2*F*_c_^2^)/3
3141 reflections	(Δ/σ)_max_ < 0.001
325 parameters	Δρ_max_ = 0.26 e Å^−^^3^
6 restraints	Δρ_min_ = −0.24 e Å^−^^3^

### Special details

**Table:**

Geometry. All e.s.d.'s (except the e.s.d. in the dihedral angle between two l.s. planes) are estimated using the full covariance matrix. The cell e.s.d.'s are taken into account individually in the estimation of e.s.d.'s in distances, angles and torsion angles; correlations between e.s.d.'s in cell parameters are only used when they are defined by crystal symmetry. An approximate (isotropic) treatment of cell e.s.d.'s is used for estimating e.s.d.'s involving l.s. planes.
Refinement. Refinement of *F*^2^ against ALL reflections. The weighted *R*-factor *wR* and goodness of fit *S* are based on *F*^2^, conventional *R*-factors *R* are based on *F*, with *F* set to zero for negative *F*^2^. The threshold expression of *F*^2^ > σ(*F*^2^) is used only for calculating *R*-factors(gt) *etc*. and is not relevant to the choice of reflections for refinement. *R*-factors based on *F*^2^ are statistically about twice as large as those based on *F*, and *R*- factors based on ALL data will be even larger.

### Fractional atomic coordinates and isotropic or equivalent isotropic displacement parameters (Å^2^)

**Table:**

	*x*	*y*	*z*	*U*_iso_*/*U*_eq_	
C1	0.17647 (17)	0.49883 (16)	−0.21123 (14)	0.0410 (5)	
H1	0.2435	0.4696	−0.2267	0.049*	
C2	0.18569 (19)	0.53311 (15)	−0.12319 (15)	0.0449 (5)	
H2A	0.1238	0.5700	−0.1085	0.054*	
H2B	0.2457	0.5757	−0.1182	0.054*	
C3	0.19786 (18)	0.44819 (14)	−0.06334 (13)	0.0403 (5)	
H3A	0.2030	0.4730	−0.0071	0.048*	
H3B	0.2625	0.4144	−0.0758	0.048*	
C4	0.10599 (15)	0.37543 (13)	−0.06804 (12)	0.0301 (4)	
C5	0.00765 (18)	0.42793 (16)	−0.03552 (15)	0.0450 (5)	
H5A	0.0050	0.4229	0.0240	0.067*	
H5B	−0.0536	0.3985	−0.0590	0.067*	
H5C	0.0104	0.4951	−0.0512	0.067*	
C6	0.09851 (15)	0.34065 (13)	−0.16103 (11)	0.0308 (4)	
H6	0.1650	0.3078	−0.1723	0.037*	
C7	0.09008 (17)	0.42290 (15)	−0.22863 (13)	0.0400 (5)	
C8	−0.0189 (2)	0.4691 (2)	−0.23163 (19)	0.0588 (7)	
H8A	−0.0280	0.5106	−0.1843	0.088*	
H8B	−0.0711	0.4191	−0.2309	0.088*	
H8C	−0.0258	0.5066	−0.2816	0.088*	
C9	0.1152 (3)	0.3832 (2)	−0.31603 (15)	0.0661 (8)	
H9A	0.1302	0.4362	−0.3528	0.099*	
H9B	0.0561	0.3476	−0.3367	0.099*	
H9C	0.1748	0.3410	−0.3130	0.099*	
C10	0.01445 (17)	0.26102 (15)	−0.16760 (12)	0.0360 (4)	
H10	−0.0525	0.2870	−0.1481	0.043*	
C11	0.04459 (17)	0.17408 (14)	−0.11425 (12)	0.0362 (4)	
H11A	0.1093	0.1471	−0.1355	0.043*	
H11B	−0.0091	0.1248	−0.1200	0.043*	
C12	0.05889 (14)	0.19643 (13)	−0.02208 (11)	0.0282 (4)	
C13	−0.05053 (15)	0.21586 (16)	0.01561 (14)	0.0406 (5)	
H13A	−0.0895	0.2574	−0.0209	0.061*	
H13B	−0.0429	0.2468	0.0687	0.061*	
H13C	−0.0870	0.1555	0.0224	0.061*	
C14	0.13479 (14)	0.28469 (13)	−0.01349 (11)	0.0279 (4)	
H14	0.2012	0.2615	−0.0364	0.033*	
C15	0.15847 (17)	0.30546 (14)	0.07854 (12)	0.0363 (4)	
H15A	0.2095	0.3574	0.0819	0.044*	
H15B	0.0950	0.3278	0.1054	0.044*	
C16	0.20003 (16)	0.21773 (15)	0.12529 (11)	0.0344 (4)	
H16	0.2676	0.1994	0.1013	0.041*	
C17	0.12547 (14)	0.13222 (13)	0.11688 (11)	0.0298 (4)	
H17	0.0574	0.1534	0.1381	0.036*	
C18	0.11087 (14)	0.10824 (13)	0.02336 (11)	0.0300 (4)	
C19	0.21614 (17)	0.07853 (14)	−0.01680 (13)	0.0385 (4)	
H19A	0.2556	0.0401	0.0218	0.058*	
H19B	0.2551	0.1357	−0.0310	0.058*	
H19C	0.2029	0.0413	−0.0661	0.058*	
C20	0.04667 (18)	0.01370 (14)	0.02911 (13)	0.0395 (5)	
H20A	−0.0254	0.0270	0.0439	0.047*	
H20B	0.0482	−0.0216	−0.0230	0.047*	
C21	0.1027 (2)	−0.04328 (15)	0.09838 (14)	0.0446 (5)	
H21A	0.1572	−0.0839	0.0751	0.054*	
H21B	0.0534	−0.0843	0.1278	0.054*	
C22	0.15043 (17)	0.03336 (14)	0.15818 (12)	0.0363 (4)	
H22	0.2265	0.0248	0.1588	0.044*	
C23	0.10904 (18)	0.01715 (16)	0.24673 (13)	0.0407 (5)	
C24	0.1500 (3)	−0.07978 (18)	0.28034 (17)	0.0605 (7)	
H24A	0.2250	−0.0802	0.2781	0.091*	
H24B	0.1231	−0.1319	0.2471	0.091*	
H24C	0.1277	−0.0879	0.3369	0.091*	
C25	−0.0109 (2)	0.0249 (2)	0.25591 (16)	0.0551 (6)	
H25A	−0.0426	0.0461	0.2042	0.066*	
H25B	−0.0407	−0.0372	0.2714	0.066*	
C26	−0.0286 (3)	0.0990 (3)	0.3237 (2)	0.0756 (9)	
H26A	−0.0801	0.0759	0.3633	0.091*	
H26B	−0.0525	0.1600	0.3004	0.091*	
C27	0.0770 (2)	0.11130 (18)	0.36501 (14)	0.0534 (6)	
H27	0.0855	0.0604	0.4070	0.064*	
C28	0.0987 (3)	0.20971 (19)	0.40561 (17)	0.0663 (8)	
C29	0.2120 (4)	0.2150 (2)	0.4350 (2)	0.0942 (13)	
H29A	0.2579	0.2094	0.3880	0.141*	
H29B	0.2255	0.1629	0.4731	0.141*	
H29C	0.2238	0.2759	0.4623	0.141*	
C30	0.0201 (4)	0.2255 (3)	0.4772 (2)	0.1030 (14)	
H30A	0.0299	0.2890	0.5004	0.155*	
H30B	0.0314	0.1775	0.5195	0.155*	
H30C	−0.0499	0.2198	0.4562	0.155*	
O2	0.21633 (15)	0.24775 (12)	0.21002 (9)	0.0491 (4)	
H2	0.2176	0.1999	0.2403	0.074*	
O3	0.00324 (17)	0.22871 (13)	−0.25149 (10)	0.0572 (5)	
H3	−0.0404	0.1852	−0.2535	0.086*	
O4	0.15864 (14)	0.57985 (12)	−0.26633 (12)	0.0549 (4)	
H4	0.2056	0.6198	−0.2605	0.082*	
O5	0.14937 (13)	0.09411 (11)	0.29913 (9)	0.0426 (4)	
O1	0.0805 (2)	0.28725 (13)	0.34886 (13)	0.0759 (7)	
H1A	0.0692	0.2651	0.3024	0.114*	

### Atomic displacement parameters (Å^2^)

**Table:**

	*U*^11^	*U*^22^	*U*^33^	*U*^12^	*U*^13^	*U*^23^
C1	0.0381 (10)	0.0370 (10)	0.0478 (11)	−0.0032 (9)	−0.0025 (9)	0.0145 (10)
C2	0.0528 (13)	0.0297 (9)	0.0523 (12)	−0.0109 (9)	−0.0068 (10)	0.0062 (9)
C3	0.0486 (11)	0.0319 (9)	0.0404 (10)	−0.0103 (9)	−0.0084 (9)	0.0051 (9)
C4	0.0328 (9)	0.0250 (8)	0.0325 (9)	−0.0007 (7)	−0.0008 (8)	0.0003 (7)
C5	0.0497 (12)	0.0345 (10)	0.0506 (12)	0.0115 (9)	0.0102 (10)	−0.0001 (9)
C6	0.0314 (9)	0.0294 (8)	0.0316 (9)	−0.0007 (8)	−0.0010 (8)	0.0027 (7)
C7	0.0443 (11)	0.0367 (10)	0.0392 (10)	−0.0067 (9)	−0.0064 (9)	0.0094 (8)
C8	0.0474 (13)	0.0524 (14)	0.0767 (18)	−0.0077 (11)	−0.0191 (13)	0.0309 (13)
C9	0.101 (2)	0.0597 (15)	0.0372 (12)	−0.0213 (16)	0.0000 (14)	0.0098 (11)
C10	0.0418 (10)	0.0342 (10)	0.0320 (9)	−0.0079 (8)	−0.0071 (8)	0.0030 (8)
C11	0.0455 (11)	0.0275 (9)	0.0356 (10)	−0.0072 (8)	−0.0075 (9)	−0.0016 (8)
C12	0.0299 (9)	0.0253 (8)	0.0294 (8)	−0.0028 (7)	−0.0015 (7)	0.0011 (7)
C13	0.0282 (9)	0.0448 (11)	0.0488 (11)	−0.0021 (8)	0.0024 (8)	0.0086 (10)
C14	0.0277 (8)	0.0264 (8)	0.0295 (8)	−0.0035 (7)	0.0004 (7)	−0.0007 (7)
C15	0.0470 (11)	0.0305 (9)	0.0315 (9)	−0.0093 (9)	−0.0028 (8)	−0.0016 (8)
C16	0.0359 (9)	0.0395 (10)	0.0279 (9)	−0.0100 (8)	−0.0030 (8)	0.0016 (8)
C17	0.0286 (9)	0.0328 (9)	0.0279 (8)	−0.0036 (7)	0.0001 (7)	0.0026 (7)
C18	0.0311 (9)	0.0272 (9)	0.0318 (9)	−0.0030 (7)	−0.0011 (8)	−0.0006 (7)
C19	0.0437 (11)	0.0328 (9)	0.0390 (10)	0.0067 (8)	0.0070 (9)	0.0011 (8)
C20	0.0494 (11)	0.0307 (9)	0.0386 (10)	−0.0109 (9)	−0.0044 (9)	−0.0005 (8)
C21	0.0600 (13)	0.0328 (10)	0.0411 (11)	−0.0061 (10)	−0.0015 (10)	0.0038 (9)
C22	0.0369 (10)	0.0344 (9)	0.0377 (10)	−0.0020 (8)	−0.0017 (8)	0.0059 (8)
C23	0.0485 (11)	0.0377 (11)	0.0359 (10)	−0.0063 (9)	−0.0050 (9)	0.0084 (9)
C24	0.091 (2)	0.0439 (13)	0.0467 (13)	−0.0027 (13)	−0.0142 (14)	0.0139 (11)
C25	0.0490 (13)	0.0685 (16)	0.0480 (12)	−0.0168 (12)	0.0038 (10)	0.0152 (12)
C26	0.0608 (17)	0.091 (2)	0.0746 (19)	0.0033 (17)	0.0135 (15)	0.0000 (19)
C27	0.0756 (17)	0.0478 (12)	0.0369 (11)	0.0024 (12)	0.0091 (11)	0.0098 (10)
C28	0.112 (2)	0.0402 (12)	0.0463 (13)	0.0050 (15)	0.0070 (15)	0.0074 (11)
C29	0.161 (4)	0.0472 (15)	0.074 (2)	−0.010 (2)	−0.046 (2)	0.0026 (15)
C30	0.171 (4)	0.067 (2)	0.071 (2)	0.009 (2)	0.040 (2)	−0.0019 (16)
O2	0.0687 (10)	0.0474 (9)	0.0311 (7)	−0.0241 (8)	−0.0123 (7)	0.0037 (7)
O3	0.0882 (13)	0.0489 (10)	0.0346 (7)	−0.0240 (9)	−0.0183 (8)	0.0033 (7)
O4	0.0598 (10)	0.0438 (9)	0.0610 (10)	−0.0155 (8)	−0.0113 (9)	0.0241 (8)
O5	0.0519 (9)	0.0414 (8)	0.0346 (7)	−0.0059 (7)	−0.0038 (6)	0.0064 (6)
O1	0.1141 (18)	0.0473 (10)	0.0663 (12)	0.0204 (12)	0.0115 (12)	0.0160 (9)

### Geometric parameters (Å, °)

**Table:**

C1—O4	1.444 (2)	C16—H16	0.9800
C1—C2	1.498 (3)	C17—C22	1.549 (3)
C1—C7	1.548 (3)	C17—C18	1.552 (2)
C1—H1	0.9800	C17—H17	0.9800
C2—C3	1.524 (3)	C18—C20	1.543 (2)
C2—H2A	0.9700	C18—C19	1.549 (3)
C2—H2B	0.9700	C19—H19A	0.9600
C3—C4	1.547 (3)	C19—H19B	0.9600
C3—H3A	0.9700	C19—H19C	0.9600
C3—H3B	0.9700	C20—C21	1.540 (3)
C4—C5	1.543 (3)	C20—H20A	0.9700
C4—C14	1.572 (2)	C20—H20B	0.9700
C4—C6	1.574 (3)	C21—C22	1.554 (3)
C5—H5A	0.9600	C21—H21A	0.9700
C5—H5B	0.9600	C21—H21B	0.9700
C5—H5C	0.9600	C22—C23	1.536 (3)
C6—C10	1.540 (3)	C22—H22	0.9800
C6—C7	1.575 (3)	C23—O5	1.450 (3)
C6—H6	0.9800	C23—C24	1.534 (3)
C7—C8	1.534 (3)	C23—C25	1.546 (3)
C7—C9	1.543 (3)	C24—H24A	0.9600
C8—H8A	0.9600	C24—H24B	0.9600
C8—H8B	0.9600	C24—H24C	0.9600
C8—H8C	0.9600	C25—C26	1.510 (4)
C9—H9A	0.9600	C25—H25A	0.9700
C9—H9B	0.9600	C25—H25B	0.9700
C9—H9C	0.9600	C26—C27	1.515 (4)
C10—O3	1.428 (2)	C26—H26A	0.9700
C10—C11	1.524 (3)	C26—H26B	0.9700
C10—H10	0.9800	C27—O5	1.427 (3)
C11—C12	1.526 (3)	C27—C28	1.531 (4)
C11—H11A	0.9700	C27—H27	0.9800
C11—H11B	0.9700	C28—O1	1.425 (3)
C12—C13	1.549 (3)	C28—C29	1.525 (5)
C12—C14	1.563 (2)	C28—C30	1.544 (5)
C12—C18	1.567 (2)	C29—H29A	0.9600
C13—H13A	0.9600	C29—H29B	0.9600
C13—H13B	0.9600	C29—H29C	0.9600
C13—H13C	0.9600	C30—H30A	0.9600
C14—C15	1.538 (3)	C30—H30B	0.9600
C14—H14	0.9800	C30—H30C	0.9600
C15—C16	1.520 (3)	O2—H2	0.8200
C15—H15A	0.9700	O3—H3	0.8200
C15—H15B	0.9700	O4—H4	0.8200
C16—O2	1.440 (2)	O1—H1A	0.8200
C16—C17	1.522 (2)		
			
O4—C1—C2	110.43 (18)	O2—C16—H16	108.6
O4—C1—C7	107.41 (16)	C15—C16—H16	108.6
C2—C1—C7	116.14 (18)	C17—C16—H16	108.6
O4—C1—H1	107.5	C16—C17—C22	120.91 (15)
C2—C1—H1	107.5	C16—C17—C18	109.06 (15)
C7—C1—H1	107.5	C22—C17—C18	104.68 (15)
C1—C2—C3	111.31 (17)	C16—C17—H17	107.2
C1—C2—H2A	109.4	C22—C17—H17	107.2
C3—C2—H2A	109.4	C18—C17—H17	107.2
C1—C2—H2B	109.4	C20—C18—C19	105.34 (16)
C3—C2—H2B	109.4	C20—C18—C17	100.71 (15)
H2A—C2—H2B	108.0	C19—C18—C17	110.87 (15)
C2—C3—C4	112.93 (17)	C20—C18—C12	117.21 (15)
C2—C3—H3A	109.0	C19—C18—C12	112.30 (15)
C4—C3—H3A	109.0	C17—C18—C12	109.77 (15)
C2—C3—H3B	109.0	C18—C19—H19A	109.5
C4—C3—H3B	109.0	C18—C19—H19B	109.5
H3A—C3—H3B	107.8	H19A—C19—H19B	109.5
C5—C4—C3	107.38 (16)	C18—C19—H19C	109.5
C5—C4—C14	112.02 (16)	H19A—C19—H19C	109.5
C3—C4—C14	108.10 (15)	H19B—C19—H19C	109.5
C5—C4—C6	114.56 (17)	C21—C20—C18	103.09 (16)
C3—C4—C6	106.87 (15)	C21—C20—H20A	111.1
C14—C4—C6	107.61 (14)	C18—C20—H20A	111.1
C4—C5—H5A	109.5	C21—C20—H20B	111.1
C4—C5—H5B	109.5	C18—C20—H20B	111.1
H5A—C5—H5B	109.5	H20A—C20—H20B	109.1
C4—C5—H5C	109.5	C20—C21—C22	106.51 (16)
H5A—C5—H5C	109.5	C20—C21—H21A	110.4
H5B—C5—H5C	109.5	C22—C21—H21A	110.4
C10—C6—C4	108.96 (15)	C20—C21—H21B	110.4
C10—C6—C7	114.70 (15)	C22—C21—H21B	110.4
C4—C6—C7	116.19 (16)	H21A—C21—H21B	108.6
C10—C6—H6	105.3	C23—C22—C17	117.05 (17)
C4—C6—H6	105.3	C23—C22—C21	109.87 (17)
C7—C6—H6	105.3	C17—C22—C21	104.55 (15)
C8—C7—C9	107.9 (2)	C23—C22—H22	108.4
C8—C7—C1	111.95 (19)	C17—C22—H22	108.4
C9—C7—C1	104.9 (2)	C21—C22—H22	108.4
C8—C7—C6	112.53 (18)	O5—C23—C24	108.09 (17)
C9—C7—C6	111.07 (19)	O5—C23—C22	108.07 (16)
C1—C7—C6	108.25 (16)	C24—C23—C22	109.6 (2)
C7—C8—H8A	109.5	O5—C23—C25	104.3 (2)
C7—C8—H8B	109.5	C24—C23—C25	111.5 (2)
H8A—C8—H8B	109.5	C22—C23—C25	114.89 (19)
C7—C8—H8C	109.5	C23—C24—H24A	109.5
H8A—C8—H8C	109.5	C23—C24—H24B	109.5
H8B—C8—H8C	109.5	H24A—C24—H24B	109.5
C7—C9—H9A	109.5	C23—C24—H24C	109.5
C7—C9—H9B	109.5	H24A—C24—H24C	109.5
H9A—C9—H9B	109.5	H24B—C24—H24C	109.5
C7—C9—H9C	109.5	C26—C25—C23	105.3 (2)
H9A—C9—H9C	109.5	C26—C25—H25A	110.7
H9B—C9—H9C	109.5	C23—C25—H25A	110.7
O3—C10—C11	108.21 (17)	C26—C25—H25B	110.7
O3—C10—C6	110.93 (17)	C23—C25—H25B	110.7
C11—C10—C6	110.19 (16)	H25A—C25—H25B	108.8
O3—C10—H10	109.2	C25—C26—C27	105.0 (2)
C11—C10—H10	109.2	C25—C26—H26A	110.7
C6—C10—H10	109.2	C27—C26—H26A	110.7
C10—C11—C12	114.79 (16)	C25—C26—H26B	110.7
C10—C11—H11A	108.6	C27—C26—H26B	110.7
C12—C11—H11A	108.6	H26A—C26—H26B	108.8
C10—C11—H11B	108.6	O5—C27—C26	103.5 (2)
C12—C11—H11B	108.6	O5—C27—C28	110.3 (2)
H11A—C11—H11B	107.5	C26—C27—C28	116.6 (3)
C11—C12—C13	107.89 (16)	O5—C27—H27	108.7
C11—C12—C14	108.52 (15)	C26—C27—H27	108.7
C13—C12—C14	113.09 (16)	C28—C27—H27	108.7
C11—C12—C18	110.36 (15)	O1—C28—C29	108.6 (3)
C13—C12—C18	109.58 (15)	O1—C28—C27	111.2 (2)
C14—C12—C18	107.41 (14)	C29—C28—C27	110.3 (3)
C12—C13—H13A	109.5	O1—C28—C30	105.4 (3)
C12—C13—H13B	109.5	C29—C28—C30	112.4 (3)
H13A—C13—H13B	109.5	C27—C28—C30	108.9 (3)
C12—C13—H13C	109.5	C28—C29—H29A	109.5
H13A—C13—H13C	109.5	C28—C29—H29B	109.5
H13B—C13—H13C	109.5	H29A—C29—H29B	109.5
C15—C14—C12	110.63 (14)	C28—C29—H29C	109.5
C15—C14—C4	115.82 (15)	H29A—C29—H29C	109.5
C12—C14—C4	115.13 (14)	H29B—C29—H29C	109.5
C15—C14—H14	104.6	C28—C30—H30A	109.5
C12—C14—H14	104.6	C28—C30—H30B	109.5
C4—C14—H14	104.6	H30A—C30—H30B	109.5
C16—C15—C14	113.40 (16)	C28—C30—H30C	109.5
C16—C15—H15A	108.9	H30A—C30—H30C	109.5
C14—C15—H15A	108.9	H30B—C30—H30C	109.5
C16—C15—H15B	108.9	C16—O2—H2	109.5
C14—C15—H15B	108.9	C10—O3—H3	109.5
H15A—C15—H15B	107.7	C1—O4—H4	109.5
O2—C16—C15	106.89 (16)	C27—O5—C23	108.82 (18)
O2—C16—C17	113.42 (15)	C28—O1—H1A	109.5
C15—C16—C17	110.66 (15)		
			
O4—C1—C2—C3	176.51 (18)	O2—C16—C17—C18	178.19 (16)
C7—C1—C2—C3	53.9 (3)	C15—C16—C17—C18	58.1 (2)
C1—C2—C3—C4	−58.2 (3)	C16—C17—C18—C20	172.77 (15)
C2—C3—C4—C5	−66.8 (2)	C22—C17—C18—C20	42.05 (18)
C2—C3—C4—C14	172.11 (17)	C16—C17—C18—C19	61.7 (2)
C2—C3—C4—C6	56.5 (2)	C22—C17—C18—C19	−69.06 (18)
C5—C4—C6—C10	−66.2 (2)	C16—C17—C18—C12	−63.00 (19)
C3—C4—C6—C10	174.97 (15)	C22—C17—C18—C12	166.28 (14)
C14—C4—C6—C10	59.06 (19)	C11—C12—C18—C20	−66.3 (2)
C5—C4—C6—C7	65.2 (2)	C13—C12—C18—C20	52.4 (2)
C3—C4—C6—C7	−53.6 (2)	C14—C12—C18—C20	175.57 (15)
C14—C4—C6—C7	−169.54 (15)	C11—C12—C18—C19	55.9 (2)
O4—C1—C7—C8	−47.7 (3)	C13—C12—C18—C19	174.53 (16)
C2—C1—C7—C8	76.5 (2)	C14—C12—C18—C19	−62.25 (19)
O4—C1—C7—C9	69.1 (2)	C11—C12—C18—C17	179.69 (15)
C2—C1—C7—C9	−166.7 (2)	C13—C12—C18—C17	−61.64 (19)
O4—C1—C7—C6	−172.26 (17)	C14—C12—C18—C17	61.57 (18)
C2—C1—C7—C6	−48.1 (2)	C19—C18—C20—C21	71.6 (2)
C10—C6—C7—C8	53.4 (3)	C17—C18—C20—C21	−43.78 (19)
C4—C6—C7—C8	−75.3 (2)	C12—C18—C20—C21	−162.75 (17)
C10—C6—C7—C9	−67.8 (2)	C18—C20—C21—C22	29.8 (2)
C4—C6—C7—C9	163.6 (2)	C16—C17—C22—C23	90.9 (2)
C10—C6—C7—C1	177.61 (18)	C18—C17—C22—C23	−145.68 (17)
C4—C6—C7—C1	49.0 (2)	C16—C17—C22—C21	−147.28 (18)
C4—C6—C10—O3	178.68 (17)	C18—C17—C22—C21	−23.9 (2)
C7—C6—C10—O3	46.5 (2)	C20—C21—C22—C23	122.8 (2)
C4—C6—C10—C11	−61.5 (2)	C20—C21—C22—C17	−3.6 (2)
C7—C6—C10—C11	166.30 (17)	C17—C22—C23—O5	−56.9 (2)
O3—C10—C11—C12	−179.57 (18)	C21—C22—C23—O5	−175.92 (17)
C6—C10—C11—C12	59.0 (2)	C17—C22—C23—C24	−174.54 (19)
C10—C11—C12—C13	71.6 (2)	C21—C22—C23—C24	66.5 (2)
C10—C11—C12—C14	−51.3 (2)	C17—C22—C23—C25	59.0 (3)
C10—C11—C12—C18	−168.72 (16)	C21—C22—C23—C25	−60.0 (3)
C11—C12—C14—C15	−175.74 (16)	O5—C23—C25—C26	−7.7 (3)
C13—C12—C14—C15	64.6 (2)	C24—C23—C25—C26	108.7 (2)
C18—C12—C14—C15	−56.43 (19)	C22—C23—C25—C26	−125.8 (2)
C11—C12—C14—C4	50.6 (2)	C23—C25—C26—C27	−13.9 (3)
C13—C12—C14—C4	−69.1 (2)	C25—C26—C27—O5	30.9 (3)
C18—C12—C14—C4	169.89 (14)	C25—C26—C27—C28	152.2 (2)
C5—C4—C14—C15	−60.1 (2)	O5—C27—C28—O1	64.9 (3)
C3—C4—C14—C15	58.1 (2)	C26—C27—C28—O1	−52.7 (3)
C6—C4—C14—C15	173.16 (15)	O5—C27—C28—C29	−55.5 (3)
C5—C4—C14—C12	71.2 (2)	C26—C27—C28—C29	−173.2 (3)
C3—C4—C14—C12	−170.70 (16)	O5—C27—C28—C30	−179.3 (3)
C6—C4—C14—C12	−55.6 (2)	C26—C27—C28—C30	63.0 (3)
C12—C14—C15—C16	54.5 (2)	C26—C27—O5—C23	−37.5 (2)
C4—C14—C15—C16	−172.13 (16)	C28—C27—O5—C23	−163.0 (2)
C14—C15—C16—O2	−178.89 (16)	C24—C23—O5—C27	−90.3 (2)
C14—C15—C16—C17	−54.9 (2)	C22—C23—O5—C27	151.15 (18)
O2—C16—C17—C22	−60.5 (2)	C25—C23—O5—C27	28.5 (2)
C15—C16—C17—C22	179.36 (16)		

### Hydrogen-bond geometry (Å, °)

**Table:**

*D*—H···*A*	*D*—H	H···*A*	*D*···*A*	*D*—H···*A*
O1—H1A···O2	0.82	2.41	2.882 (3)	117
O2—H2···O5	0.82	1.95	2.697 (2)	152
O3—H3···O4^i^	0.82	2.12	2.929 (3)	169
O4—H4···O2^ii^	0.82	2.13	2.890 (2)	153

Symmetry codes: (i) −*x*, *y*−1/2, −*z*−1/2; (ii) −*x*+1/2, −*y*+1, *z*−1/2.
